# Low-Carbohydrate Ketogenic Diets in Male Endurance Athletes Demonstrate Different Micronutrient Contents and Changes in Corpuscular Haemoglobin over 12 Weeks

**DOI:** 10.3390/sports7090201

**Published:** 2019-08-30

**Authors:** Fionn T. McSwiney, Lorna Doyle

**Affiliations:** 1 Department of Sport and Exercise Science, Waterford Institute of Technology, X91KOEK Waterford, Ireland; 2 School of Health and Human Performance, Dublin City University, Glasnevin, 9 D09V209 Dublin, Ireland

**Keywords:** high-carbohydrate, ketogenic, diet, micronutrient, haematology, endurance, athletes

## Abstract

High-carbohydrate (HC) diets and low-carbohydrate ketogenic diets (LCKD) are consumed by athletes for body composition and performance benefits. Little research has examined nutrient density of self-selected HC or LCKDs and consequent effect on blood haematology in an athlete population. Using a non-randomised control intervention trial, nutrient density over 3 days, total blood count and serum ferritin, within endurance athletes following a self-selected HC (n = 11) or LCKD (n = 9) over 12 weeks, was examined. At week 12, HC diet participants had greater intakes of carbohydrate, fibre, sugar, sodium, chloride, magnesium, iron, copper, manganese and thiamine, with higher glycaemic load (GL), compared to LCKD participants (*P* < 0.05). LCKD participants had greater intakes of saturated fat, protein, a higher omega 3:6 ratio, selenium, vitamins A, D, E, K_1_, B_12_, B_2_, pantothenic acid and biotin. Mean corpuscular haemoglobin (MCH) and mean corpuscular haemoglobin concentration (MCHC) decreased in LCKD participants after 12 weeks but remained unchanged in HC participants, with no change in serum ferritin in either group. This analysis cannot examine nutrient deficiency, but athletes should be made aware of the importance of changes in dietary type on micronutrient intakes and blood haematology, especially where performance is to be considered.

## 1. Introduction

The LCKD has emerged as a popular method for decreasing body mass in overweight patients [1], improving body composition in athletic populations [2,3,4] and has been examined in terms of exercise performance effects [5,6,7]. The ketogenic diet, initially used as a treatment in children with drug-resistant epilepsy [8,9] has been examined in other clinical settings, such as neuromuscular and neurodegenerative diseases [10,11], and type-2 diabetes mellitus [1,12]. Despite the broad range of research examining its effects in non-athletic and athletic populations, little research has examined the micronutrient density of an LCKD.

A recent review by Churuangsut et al. [13] reported reduced intakes of thiamine, folate, magnesium, calcium, iron and iodine upon consumption of carbohydrate-restricted diets, but no study reviewed was classed as “ketogenic”. In addition, several trials presented within the review [13] were weight loss trials, so decreased micronutrient consumption was expected. A hypothetical case study calculated Australian males and females could exceed the minimum nutrient intakes for all micronutrients on a low-carbohydrate high-fat (LCHF) diet, containing ~80 g/d carbohydrate [14]. Ketogenic diets and LCHF diets are similar as both restrict carbohydrates and increase dietary fat; however, it may be more difficult for an individual following an LCKD to meet nutrient guidelines, such as fibre and thiamine, due to the level of carbohydrate restriction (<50 g/d), required for “nutritional ketosis” [15] versus an LCHF diet (~80–130 g/d carbohydrate) [14,16]. Taylor et al. [17], upon examination of micronutrient density of an LCKD through 3-day food records, found consumption of an LCKD increased vitamin K and choline intakes from insufficient to sufficient levels, while manganese decreased to insufficient levels. 

Changes in micronutrient intake upon LCKD consumption could affect blood haematology. There is limited research on blood haematology changes upon LCKD consumption. Kose et al. [18] found drug-resistant epilepsy patients treated for 6 months with a LCKD demonstrated increases in several haematological parameters, including haemoglobin, haematocrit, MCV and serum vitamin B_12_, but suggested further research is vital to confirm such findings. Within athletes, exercising under low carbohydrate availability, has been suggested to influence the iron regulatory hormone hepcidin, and increase inflammation [19]. Increased inflammation is associated with increased ferritin levels [20]. Recent research reported no difference in pre-exercise inflammation (interleukin-6 (IL-6) following 3 weeks of an LCHF or HC diet consumption, in international-level race walkers [21]. Additionally, despite increased iron intake within HC participants, pre-exercise serum ferritin and haemoglobin were significantly higher following LCHF diet consumption. However, post-exercise IL-6 was significantly higher within the LCHF group, with the HC group experiencing an attenuated post-exercise hepcidin response. This was suggested to be due to higher pre-exercise serum ferritin within the LCHF group [21]. With blood haematology being an indicator of endurance capacity [22], it is vital whatever diet is adhered to ensures cardiovascular endurance capacity. 

The primary aim of this research was to compare nutrient intakes of endurance athletes following a traditional HC diet versus an LCKD during a 12-week dietary and training intervention. With micronutrient intakes having the potential to affect blood haematological parameters and hence, athlete performance, a secondary aim was to examine how HC or LCKD consumption over 12 weeks, affected pre-exercise blood haematology. 

## 2. Materials and Methods

Details of this investigation have been described previously [3]. A minimum sample size of 9 participants per group was calculated using G*Power version 3.1.9.4. (Universität Düsseldorf, Düsseldorf, Germany), with reference to dietary intake data reported in similar ketogenic research [23]. In brief, 47 male endurance athletes aged 19–40 years participated in a 12-week non-randomised control trial, comparing performance. All participants competed in endurance events (>2 years), were 18–40 years of age and habitually consumed a HC diet (~50% of total calories). Twenty participants completed all aspects of the dietary and training intervention [3], whilst a total blood count was performed on 19 participants (HC *n* 10, LCKD *n* 9), due to insufficient blood sample from 1 participant. All procedures were approved by the research ethics committee at Waterford Institute of Technology and all participants provided written informed consent. The study is registered with the ISRCTN registry as number ISRCTN70987680 (direct link to registered study information: https://doi.org/10.1186/ISRCTN70987680).

### 2.1. Dietary Intervention

Food diaries were obtained at baseline using a 3-day weighed food diary (2 week days and 1 weekend day). These were analyzed using Nutritics dietary analysis software (Nutritics Professional v3.09, Nutritics, Dublin, Ireland). The macronutrient goals were: HC 65% carbohydrate, 20% fat and 14% protein or LCKD >75% fat, 10–15% protein and <50 g/d carbohydrate. At the outset, participants in each group received a nutritional handout, detailing how best to formulate their diet according to daily energy requirements. Participants in each group received nutritional counselling throughout the 12-week intervention. Example meal plans for the HC and LCKD groups are detailed in Table 1.

Participants were contacted weekly to ensure dietary adherence and a weighed food diary was submitted each week. Three-day food diaries were also obtained in week 12 and analysed using the Nutritics dietary analysis software. Average nutrient intake, including GL and potential renal acid load (PRAL) for 3 days pre- and post-dietary intervention were obtained from the dietary analysis report. 

### 2.2. Blood Analysis

Fasting blood samples (following 48 h rest from training) were collected into an ethylenediaminetetraacetic acid (EDTA) tube from an antecubital vein using a 21G needle (BD Diagnostics, Dublin, Ireland) at pre- and post-intervention testing, subsequent to a 12-h overnight fast. Blood samples were put through a haematology analyser (Beckman Coulter AcT diff Analyzer, Beckman Coulter, Brea, CA, USA) 20–30 min subsequent to collection. The haematology analyser measured participant’s white blood cells, lymphocytes, monocytes, granulocytes, red blood cells, haematocrit, mean corpuscular volume (MCV), mean corpuscular width (MCW), red blood cell distribution width (RDW) and platelet content. Serum ferritin was measured pre and post intervention via Enzyme Linked Immunosorbent Assay (ELISA) (GmbH, Aachen, Germany). Intra-assay coefficient of variation was 3.9–9.9 ng/mL.

### 2.3. Net Endogenous Acid Production

Net endogenous acid production (NEAP) was quantified using protein and potassium intakes at pre- and post-intervention testing, using two algorithms [24].

(1) Estimated NEAP_1_ (mEq∙day^−1^) = [0.91 × protein (g∙day^−1^)] − [0.57 × potassium (mEq∙day^−1^)] + 21
(2) Estimated NEAP_2_ (mEq∙day^−1^) = [54.5 × protein (g∙day^−1^)/potassium (mEq∙day^−1^)] − 10.2

### 2.4. Statistical Analysis

IBM Statistics SPSS 24 (Illinois, Chicago, IL, USA) was used for statistical analysis. Data were tested for normality with the 1-sample Kolmogorov–Smirinoff test. Parametric tests were used for normally distributed data or non-parametric for data not normally distributed. Independent sample *t*-tests or Mann–Whitney U test (if data was not normally distributed) were used to determine differences between HC and LCKD groups at baseline, with the alpha level for significance set at *P* < 0.05. Analysis of Covariance (ANCOVA) was used for statistical analysis at post-intervention testing, with pre-intervention measures acting as a covariate. Paired samples *t*-tests or Wilcoxon signed ranks test (if data was not normally distributed) examined changes over time within each group, if the ANCOVA *P* value was <0.05. 

## 3. Results

### 3.1. Subject Characteristics

Baseline subject characteristics presented in Table 2 [3] demonstrate no difference in age, height or BMI between HC and LCKD participants.

### 3.2. Nutrient Changes over Intervention Period

Table 3 presents mean nutrient intake for HC and LCKD groups, as well as recommended daily allowances (RDAs) for European citizens [25], from the World Health Organisation (WHO) [26,27] and for endurance athletes [28,29]. No changes in intake of energy, potassium, calcium, phosphorus, zinc, niacin, folic acid, vitamin C, potential renal acid load (PRAL) or caffeine took place across the intervention period. As expected, carbohydrate consumption decreased and fat increased in the LCKD group, causing a reduction in GL. Protein remained unchanged in the HC group and increased in LCKD participants across the intervention period. 

Additional energy from dietary fat resulted in increases in energy from saturated fat in the LCKD group, while the HC group reduced saturated fat intake. Omega 3:6 ratio remained unchanged within HC participants, but increased within LCKD participants. Intake of fibre, free sugar, sodium, chloride, magnesium, iron, copper and thiamine decreased within LCKD participants, but was unchanged in HC participants. Intakes of selenium, vitamin A, D, E, K_1_, B_12_, riboflavin and biotin increased within LCKD participants. ANCOVA demonstrated a significant difference in post-intervention intakes of pantothenic acid and vitamin B_6_, with higher intakes within the LCKD group. Within HC participants, intake of manganese increased while intakes of iodine and vitamins A, D, riboflavin and B_12_ decreased with increased carbohydrate focus within the diet.

### 3.3. Net Endogenous Acid Production (*NEAP*)

Table 4 presents NEAP values for HC and LCKD groups. There were no differences between groups NEAP_1_ and NEAP_2_ at baseline. Consumption of an LCKD increased NEAP_1_ (*P* = 0.038) and caused a difference between groups at post-intervention testing (*P* = 0.041). No changes in NEAP_2_ were observed. 

### 3.4. Blood Analysis

Table 5 presents changes in blood measures for HC and LCKD groups. MCH and MCHC both decreased in the LCKD group. No other significant changes were observed, but a medium effect size in RDW occurred within LCKD.

## 4. Discussion

Examination of nutrient density within endurance athletes who adhered to a HC or LCKD revealed consumption of a HC diet resulted in greater intakes of fibre, sugar, sodium, chloride, magnesium, iron, copper, manganese and thiamine, with higher GL, compared to LCKD consumption. Conversely, free-living consumption of a LCKD resulted in greater intake of saturated fat, protein, a higher omega 3:6 ratio and greater intakes of selenium, vitamins A, D, E, K_1_, riboflavin, pantothenic acid, B_12_, B_6_, biotin and higher NEAP. Blood analysis revealed consumption of a LCKD for 12 weeks reduced MCH and MCHC amongst LCKD participants, but no change in iron stores were evident. 

The American College of Sports Medicine advocates a nutrient dense HC diet with appropriate energy for optimal sports performance and health amongst endurance athletes [29]. However, metabolic adaptations such as decreased reliance on carbohydrates [3,5,23,31], and improvements in power to weight ratio [3,7] have made a LCKD a desirable approach for some endurance athletes. 

Despite the HC group within this research increasing carbohydrate intake, levels were still marginally below the 6–12 g·kg recommended range for endurance athletes exercising 1–3 h a day [28]. This is contrary to a review which found male athletes generally achieve their carbohydrate recommendations [32]. Notably, however, Burke et al. [32] included competition dietary intakes where energy and carbohydrate requirements are greater. In contrast, participants’ food diaries within the current investigation were obtained during the final week of a training intervention, when participants’ completed 11.7 ± 1.7 h of endurance training/week [3]. Another factor which likely contributed to sub-optimal carbohydrate intake was prioritisation of whole foods, resulting in a high fibre intake. Gastrointestinal limits to bulky, high-fibre foods, such as whole grains products and potatoes, are reported to impede optimal carbohydrate intakes [28]. By design, carbohydrate intake within the LCKD group was significantly beneath this threshold (0.5 g·kg) in order to induce a state of nutritional ketosis (i.e., elevate blood ketones to >0.5 mmol/L) [15]. When adopting an LCKD, individuals exclude many foods high in fibre, such as cereals and wholegrain products. However on a well-formulated LCKD [15], green leafy fibrous vegetables, nuts, seeds and low-carbohydrate fruits such as avocados are nutritious sources of fibre, and should be encouraged to prevent sub-optimal fibre intake, observed upon LCKD consumption within the current research. 

Post-intervention protein intake significantly differed between groups, with protein intake within the HC group slightly below the 1.4–2.0 g·kg range recommended for endurance athletes [33]. However, neither group experienced loss of lean body mass (lean body mass: HC = +0.1 kg and LCKD = +0.3 kg, *P* > 0.05, ES: 0.068) [3], which is a good indication each group was consuming adequate energy and protein across the intervention period. 

Dietary fat increased to 77.2% energy intake within LCKD participants and non-significantly decreased to 18.6% energy intake within HC participants. Following a popular publication in lay press [15], this investigation encouraged consumption of foods containing monounsaturated (e.g., nuts, avocados, olive oil) and saturated fats (e.g., fatty animal meat, butter, coconut oil) and less so foods containing polyunsaturated fats (e.g., soy and corn oil). This was reflected within participants reported diets, as monounsaturated (34.2% total energy) and saturated fat (29.5% total energy) comprised ~63.7% of total energy. Long-term high saturated fat intake is an important consideration in nutrition. A robust relationship between saturated fat and health is not settled in science [34]. Participants within the current research were endurance athletes, consuming an LCKD. These saturated fatty acids derived from butter and coconut oil are rich in short- and medium-chain fatty acids, which have been suggested to be easier to oxidize compared to long-chain fatty acids [35], hence high saturated fat intake in the context of an LCKD within an endurance athlete population, may not be problematic [36]. 

Increases in saturated fat occurred in the presence of increased polyunsaturated fatty acid intake, within LCKD participants, with improved omega 3:6 ratio. A low omega 3:6 ratio is pro-inflammatory and thought to accelerate pathogenesis of chronic diseases, such as CVD, cancer and autoimmune diseases [37,38]. Hence, an increased omega 3:6 fatty acid ratio within the LCKD is desirable. Within HC participants, increased emphasis should be placed on the importance of polyunsaturated and monounsaturated fats to improve omega 3:6 ratios, and achieve appropriate fat recommendations within endurance athletes [39]. 

Thiamine consumption decreased in the LCKD group, but was still above the recommended intake threshold. This was not surprising, as wholegrain foods are the predominant sources. However, as outlined by Zinn et al. [14] in the context of a low-carbohydrate high-fat diet containing ~80 g/d of carbohydrates, one must question if an individual’s thiamine requirements are reduced, as one of the key functions of thiamine is the metabolism of carbohydrates [40]. It is important athletes consuming a ketogenic diet are aware of the importance of consuming thiamine-rich foods.

During our assessment, LCKD participants had greater intakes of Vitamins A, D, K_1_, pantothenic acid, biotin and vitamin B_12_ compared to HC participants. Vitamin D insufficiency is common in athletic populations and dietary interventions are often unsuccessful at remedying deficiencies [41], although the current participants upon LCKD consumption achieved sufficient dietary Vitamin D intake. Average intakes of vitamin B_12_ range from 4.2–8.6 µg/d in adults; however, intakes in males are higher in a number of European countries [25], but not considered harmful [42]. Although only a snapshot of HC participants diets, vitamin A and B_12_ consumption within the current group was below recommended levels for European males [25]. Individuals following a HC diet should be advised to keep dietary fat ~20–30% of total energy, with sufficient diet variety, to ensure a diverse range of vitamins. A recent position stand from the American College of Sports Medicine stated there is no performance benefit to consuming <20% of total energy from fat [29]. 

Electrolyte balance is an important consideration when working with any athlete; however, when following an LCKD, it is paramount that athletes take additional measures to ensure adequate hydration, as the absorption of glucose across the intestinal wall is dependent on sodium, with the involvement of a sodium/glucose co-transporter [43]. Low-carbohydrate experts recommend consuming ~5000 mg/d of sodium on an LCKD to preclude orthostatic symptoms [15]. Therefore, LCKD participants were recommended to supplement sodium at meal times (i.e., table salt), consume electrolytes when exercising and consume 1–2 g/d of sodium from bouillon cubes or homemade broth [3]. Additionally, LCKD participants were advised to salvage nutrient-dense meat drippings when cooking to garnish dishes and recommended to grill meat and steam vegetables, as magnesium and other nutrients can be spoiled through boiling/evaporation [15]. Despite these measures, LCKD participants reported sodium intake was sub optimal, while they consumed adequate potassium and magnesium. Orthostatic symptoms were not reported to the researcher subsequent to the first 0–2 weeks of the intervention, when participants’ were still developing new dietary routines and physiologically adapting to their new lifestyle. Thus, LCKD participant’s daily intake of sodium was sufficient to preclude orthostatic symptoms in trained individuals in the current research. It is most likely that LCKD participants poorly reported their added salt (sodium) at mealtimes, most probably underestimating or omitting discretional salt intake. Appropriate sodium, potassium and magnesium intakes are necessary to achieve water balance and maintain nerve and muscle function [15], and vital in endurance athletes when electrolyte balance is threatened due to sweating. Phosphorus, magnesium, iron and copper were consumed in significantly higher amounts by HC participants with lower amounts consumed by the LCKD group. 

Iron deficiencies, even in the absence of anaemia can impair muscle function [44], which in an athletic population can attribute to poor work capacity and poor training adaptations [29]. Iron intakes met the requirements for the general population in each group, but decreases observed within the LCKD meant their intake was below the RDA specific for endurance athletes (~19 mg/d) [29]. This is similar to previous research reporting reduced iron intake in an LCHF diet compared to a HC diet amongst athletes [21]. A well-formulated ketogenic diet, including organ meat, red meat, eggs and green leafy vegetables should provide sufficient iron intake, hence the participants within the current investigation may not have consumed a sufficient variety of these advised foods. Of additional concern, LCKD participants observed decreases in copper intake. A recent article highlighted the development of copper-deficiency anaemia in a child transitioning from a formula-based LCKD to a pureed food-based LCKD, with the deficiency being rectified by copper supplementation [45]. Similar to iron, reduced variety in food intake may have resulted in reduced copper intake within LCKD participants, as such it is paramount this aspect is emphasized to anyone adopting an LCKD. Organ meat, nuts, soy-products, beans and sea-food are all high in copper, so when included within a LCKD should provide sufficient intake. Post-intervention, the HC groups haemoglobin count was >13.0 g/dL, a recommended threshold set for healthy males [46]. In contrast, post-intervention haemoglobin was below the ~13 mg/dL threshold within the LCKD group, placing LCKD participants marginally above the mild anaemia threshold (i.e., 11–12.9 g/dL) [46]. This is in contrast to Kose et al. [18], where increases in haematological parameters were observed upon LCKD consumption, but participants were not endurance athletes, nor was iron intake of study participants reported. McKay et al. [21] reported increased pre-exercise haemoglobin levels within athletes, upon LCHF diet consumption in comparison to a HC diet. However, this research was 3-weeks long [21] compared to the current study which was 12 weeks. In addition, LCKD participants’ experienced decreases in MCH and MCHC but remained within the normal range; however, haematocrit concentrations in each group were low. These findings are not uncommon among trained individuals, Sharpe et al. [47] found that 85% of females and 22% of males in a cohort of >1000 people had haematocrit of <0.42. Additionally, vitamin C intake within the LCKD group was 21.8% below the recommended daily intake, as a LCKD is limited in fruit intake. Ascorbic acid has a dose-dependent enhancing effect on iron absorption in humans [48]. In light of low haemoglobin/haematocrit count and decreased MCH and MCHC within the LCKD, further analysis examined whether decreased iron impacted serum ferritin concentrations. There is no agreed definition as to the concentration of serum ferritin which constitutes a problematic level of iron depletion, but expert opinions range from <10 to <35 ng/mL [29,49]. Despite aforementioned concerns, serum ferritin concentrations remained unchanged within the LCKD group, with concentrations of serum ferritin in each group being considerably greater than deficiency thresholds previously outlined [29,49]. In response to exercise, serum ferritin may increase through the inflammatory response [50]. However, inflammation is thought to not play a role within the current investigation, since blood samples were taken 48 h after rest. Previous research has demonstrated similar pre-exercise IL-6 levels following LCHF or HC diet consumption [21]. Zinn et al. [14] highlighted that iron bioavailability is reduced by phytates, given a LCKD is free of foods high in phytates, such as wholegrains, it is not inconceivable to postulate that persons following an LCKD could have increased iron bioavailability. However, likewise, it is possible 12-weeks exercise training on a KD could have resulted in increased inflammation, in comparison to HC diet consumption, as previously reported [21], which could have affected the iron regulatory hormone hepcidin [21], reducing iron status over time. Similar to the conclusion drawn by McKay et al. [21], this is speculative, with further research required to understand the process by which a LC diet could affect iron status in athletes. 

Interest in PRAL and NEAP experienced a renaissance when NEAP calculations were included in a recent manuscript in elite endurance athletes [47]. Similar to the investigation by Carr et al. [51], NEAP_1_ increased following a period of keto-adaptation and was greater than the HC groups estimated NEAP_1_ score at post-intervention testing. Unlike Carr et al. [51], investigation, blood bicarbonate and pH were not measured, but participants contained within the current investigation had similar PRAL scores at post-intervention testing and previously experienced similar blood lactate responses to the HC group throughout a 100 km TT and subsequent to a maximal exercise test [3]. An investigation in ultra-marathon runners demonstrated that participants experienced simultaneous increases in blood pH and lactate during a 100 km running trial [52]. This investigation lacks the specificity of Carr et al. [51]; however, one could postulate based on PRAL scores and the homogeneity of HC and LCKD participants lactate responses during exercise [3] that non-elite-endurance-trained individuals may possess the same “blood buffering capacity” as elite athletes following a 12-week adaptation to an LCKD, although a more thorough investigation which examines changes in blood bicarbonate and pH is required. 

This investigation has a number of limitations, namely (1) small sample size, (2) findings are limited to the population sample at hand (i.e., male endurance athletes), (3) findings may be limited to the geographical and cultural location (i.e., Ireland), (4) this analysis is based on nutrient intakes reported over 3 days, for each time-point. Although judgments of deficiency and inadequacy cannot be made from such acute assessments (e.g., HC post Zn, I, vitamin A; LCKD post Zn, Ca), particularly when evidence is provided by dietary recall/food diaries [53], long-term implications of such intakes are an important consideration. (5) dietary analysis software may lack the delicacy to identify nutrients lost or salvaged through various cooking methods (i.e., grilling versus boiling). 

## 5. Conclusions and Recommendations

Noteworthy findings were the LCKD groups reported reduced iron intake and reduced MCH and MCHC, but maintenance of serum ferritin. This may re-highlight a limitation previously outlined (accuracy of food diaries) or perhaps, that iron bioavailability is increased on a LCKD. Irrespective of dietary approach, endurance athletes should be made aware of the dangers of low iron and encouraged to monitor blood work with a medical professional. In an attempt to increase their carbohydrate intake, HC participants restricted dietary fat to <20% of total energy, with low intakes of essential vitamins, namely vitamin A, D, E and vitamin B_12_. Therefore, individuals following a HC diet should be advised to keep dietary fat to >20–30% of total calories and to prioritise polyunsaturated and monounsaturated fatty acids to ensure a diverse range of fat-soluble vitamins. Additionally, nutritional guidelines recommend macronutrient intakes (g·kg) to optimise performance [28,29,54]. Although not always the case [29], some editions of nutritional guidelines recommend “nutrient-rich carbohydrate foods” but fail to stipulate forms [28,29]. These guidelines may be taken out of context, which may lead athletes to overly concentrate on energy and macronutrient intake (g·kg) and to prioritise carbohydrate-dense foods such as pastas, breads and cereals, which lack the micronutrient density of less calorically and carbohydrate-dense foods such as fruits and green leafy fibrous vegetables, as observed within the current investigation. Therefore, athletes adhering to a HC diet should be reminded to consume a diet with appropriate micronutrient density [25], as well as adhering to sports-specific macronutrient guidelines [28,29,54]. Finally, athlete-specific vitamin and mineral recommendations have not been designed. The current research utilizes the majority of nutrient recommendations designed for the general population. Considering the extremes athletes push themselves for performance, development of further athlete-specific micronutrient recommendations may be warranted.

## Figures and Tables

**Table 1:** Example meal plans for HC and LCKD groups.

Group	Breakfast	Snack	Lunch	Dinner	Other
HC	80 g granola, 135 mL semi-skimmed milk, 1 med apple, 1 med orange, 1 med banana, 6oz Americano, 30 mL semi-skimmed milk	2 tbsp natural yoghurt, 20 g almond butter, 1 med banana	2 cups raw baby spinach, ½ avg. avocado, 90 g chicken, 335 g sweet potato, 70 g cherry tomatoes, 35 g beetroot	80 g white fish, 405 g new potatoes, 2 tsp low-fat butter, 85 g broccoli boiled in unsalted water, 90 g steamed baby carrots	0–1 scoop whey protein (containing 20–25 g protein) and/or 0–4 energy gel(s) (containing 20–30 g of carbohydrate)
LCKD	4 avg eggs scrambled, with 1 tbsp full-fat butter, 1 tbsp full-fat cream, 2 cups of fried baby spinach, 1 cup of fried kale, 1 tsp chia seeds	6 oz. Americano, 1 tbsp full-fat butter, 1 tbsp coconut oil	100 g salmon, 1 avg avocado, 10 g blueberries, 40 g raspberries, 30 g mixed nuts, 1 tbsp coconut oil, 2 tbsp olive oil	120 g sirloin steak, 1.5 cups steamed broccoli, 2 cups steamed cauliflower, 2 tbsp full-fat butter, 2 tbsp olive oil	135 g Greek yoghurt, 1 tbsp cream, 2 avg squares 85–90% dark chocolate, 40 g raspberries and bouillon cubes or homemade broth

Abbreviations: Med = medium, tbsp = tablespoon, tsp = tea spoon, avg = average, oz = ounces.

**Table 2:** Baseline subject characteristics of HC and LCKD groups [3].

Subject Characteristics	HC Diet (*n* = 11)		LCKD (*n* = 9)		*t*-Test
	Mean ± SD	Range	Mean ± SD	Range	*P* Value
Age, years	32.1 ± 6.4	20.0–38.0	33.8 ± 6.9	19.0–40.0	0.566
Height, cm	181.2 ± 4.9	177.0–192.1	183.1 ± 5.5	175.5–191.6	0.408
BMI, kg/m^2^	23.9 ± 2.9	20.0–30.5	25.6 ± 3.0	22.2–31.3	0.090

**Table 3:** Nutrient intake for HC and LCKD groups with Recommended Intakes/Dietary Reference Values (DRV).

Nutrient	HC Diet (*n* = 10)		LCKD (*n* = 9)			
	Pre	Post	Pre	Post	ANCOVA	Recommended Intakes/DRV
	Mean ± SD ^a^	Mean ± SD	Mean ± SD ^a^	Mean ± SD	*P* Value	
Energy, kcal	2366 ± 774	2672 ± 363	2843 ± 558	3022 ± 911	0.66	NA
Carbohydrates, g·kg	3.9 ± 1.3	5.3 ± 1.4 ^c^	5.2 ± 1.3 ^b^	0.5 ± 0.1 ^c^	0.000 *	6–10 ^d^
Protein, g·kg	1.5 ± 0.4	1.2 ± 0.2	1.2 ± 0.2	1.6 ± 0.3 ^c^	0.031 *	1.2–1.7 ^e^
Fat, %/kcal	29.2 ± 13.4	18.6 ± 3.7	20.4 ± 12.3	77.2 ± 24.8 ^c^	0.000 *	20–35% ^e^
Saturated Fat, % kcal	12.4 ± 6.2	5.6 ± 2.2 ^c^	7.8 ± 1.5	29.5 ± 9.1 ^c^	0.000 *	ALAP ^f^
Omega 3:6 ratio	1:5.1	1:6.0	1:10	1:2.7 ^c^	0.004 *	NA ^f^
Fibre, g	42.8 ± 10.1	56.0 ± 19.8	48.3 ± 9.0	19.2 ± 4.9 ^c^	0.000 *	25 ^f^
Free sugars, % kcal	2.5 ± 3.8	2.2 ± 1.7	2.2 ± 2.9	0.7 ± 0.3 ^c^	0.021 *	<10 ^g^
Sodium, mg	2802 ± 1344	3714 ± 1352	3403 ± 1051	1713 ± 1100 ^c^	0.006 *	<2000 ^h^
Potassium, mg	4301 ± 1495	3650 ± 497	4674 ± 1373	4166 ± 729	0.49	3500 ^f^
Chloride, mg	4697 ± 2519	7618 ± 3176	4983 ± 3047 ^b^	2181 ± 622 ^c^	0.001 *	NA
Calcium, mg	1223 ± 424	1030 ± 278	1154 ± 352	928 ± 240	0.71	950 ^f^
Phosphorus, mg	2023 ± 565	2103 ± 460	2102 ± 422	1838 ± 379	0.26	550 ^f^
Magnesium, mg	437 ± 121	513 ± 125	509 ± 124	380 ± 39 ^c^	0.017 *	350 ^f^
Iron, mg	18.8 ± 6.9	18.2 ± 5.4	18.7 ± 4.2	12.0 ± 2.0 ^c^	0.012 *	11 ^f^
Zinc, mg	18.2 ± 13.1	13.4 ± 2.2	14.3 ± 4.2	14.4 ± 3.7	0.81	16.3 ^f^
Copper, mg	1.7 ± 0.7	1.9 ± 0.4	1.6 ± 0.3	1.3 ± 0.1 ^c^	0.011 *	1.6 ^f^
Manganese, mg	6.0 ± 2.2	10.1 ± 3.7 ^c^	8.0 ± 2.5	2.3 ± 0.4 ^c^	0.000 *	3.0 ^f^
Selenium, µg	63.9 ± 23.9	56.6 ± 15.0	39.3 ± 20.6 ^b^	136.3 ± 71.2 ^c^	0.017 *	70 ^f^
Iodine, µg	209 ± 151	87 ± 41 ^c^	185 ± 67	250 ± 108	0.000 *	150 ^f^
Vitamin A, µg	1565 ± 1335	581 ± 450 ^c^	714 ± 637 ^b^	1849 ± 558 ^c^	0.014 *	750 ^f^
Vitamin D, µg	5.4 ± 5.2	3.5 ± 5.2 ^c^	2.7 ± 2.4	17.6 ± 4.5 ^c^	0.000 *	15 ^f^
Vitamin E, mg	13.9 ± 10.3	8.6 ± 8.6	8.7 ± 7.7	23.7 ± 4.7 ^c^	0.000 *	13 ^f^
Vitamin K_1_, µg	103.3 ± 100	101.4 ± 115	34.1 ± 62 ^b^	301.3 ± 174 ^c^	0.017 *	70 ^f^
Thiamine, mg/MJ	0.21 ± 0.06	0.22 ± 0.04	0.23 ± 0.05	0.11 ± 0.04 ^c^	0.000 *	0.1 ^f^
Riboflavin, mg	2.0 ± 0.9	1.2 ± 0.4 ^c^	2.3 ± 0.6	2.8 ± 0.7 ^c^	0.000 *	1.6 ^f^
Niacin, mg NE/MJ	5.7 ± 2.0	4.6 ± 1.4	4.9 ± 1.9	4.5 ± 0.7	0.52	1.6 ^f^
Pantothenic Acid, mg	8.0 ± 3.6	6.1 ± 1.2	8.9 ± 3.2	10.2 ± 1.7	0.000 *	5.0 ^f^
Vitamin B_6_, mg	2.7 ± 0.8	3.2 ± 0.3	3.2 ± 0.9	3.6 ± 0.8	0.001 *	1.7 ^f^
Folic Acid, µg DFE	379 ± 145	311 ± 75	318 ± 164	309 ± 68	0.34	330 ^f^
Vitamin B_12_, µg	5.4 ± 2.5	2.9 ± 2.3 ^c^	4.9 ± 2.3	13.5 ± 4.5 ^c^	0.000 *	4.0 ^f^
Biotin, µg	45.0 ± 16.1	46.5 ± 14.6	50.3 ± 24.1	70.4 ± 14.6 ^c^	0.001 *	40 ^f^
Vitamin C, mg	143 ± 85	123 ± 71	134 ± 84	86 ± 68	0.21	110 ^f^
GL	146 ± 50	200 ± 86	216 ± 62 ^b^	6 ± 3 ^c^	0.000 *	NA ^f^
PRAL	25.4 ± 34.9	37.1 ± 17.1	10.0 ± 18.0	22.9 ± 27.2	0.161	NA
Caffeine, mg	111.6 ± 66.9	149.6 ± 65.8	113.3 ± 104.8	162.6 ± 113.0	0.68	NA

Abbreviations: MJ = mega-joule, NE = niacin equivalents, DFE = dietary folate equivalents, ALAP = as low as possible, NA = not applicable. ^a^ Original means and standard deviations. * ANCOVA significant difference at *P* < 0.05. ^b^ Significant difference between groups at baseline. ^c^ Significant difference (*P* < 0.05) within group between pre- and post-intervention testing. ^d^ [24]. ^e^ [25]. ^f^ [21]. ^g^ [22]. ^h^ [23].

**Table 4:** Net endogenous acid production (NEAP) for HC and LCKD groups using two algorithms [24].

Measure	HC Group (*n* = 10)		LCKD Group (*n* = 9)	
	Pre	Post	Pre	Post
	Mean ± SD	Mean ± SD	Mean ± SD	Mean ± SD
NEAP_1_, mEq∙day^−1^	64.9 ± 27.3	52.8 ± 20.9	53.0 ± 16.7	79.0 ± 30.1 *,^†^
NEAP_2_, mEq∙day^−1^	52.7 ± 19.2	44.8 ± 14.8	42.3 ± 13.1	57.4 ± 16.9

* Significant difference (*P* < 0.05) within groups between pre and post-intervention testing. ^†^ Significant difference (*P* < 0.05) between groups at post-intervention testing.

**Table 5:** Haematology results for HC and LCKD groups.

Blood Measures	HC Group (*n* = 10) ^a^			LCKD Group (*n* = 9) ^a^					
	Pre	Post	Change	Pre	Post	Change	ANCOVA		
	Mean ± SD	Mean ± SD	Mean	Mean ± SD	Mean ± SD	Mean	F-value	*P* value	ES ^b^: *N*p^2^
RBC, 10^−6^ µL	4.59 ± 0.33	4.30 ± 0.63	−0.29	4.67 ± 0.42	4.44 ± 0.61	−0.23	(1,17) = 0.074	0.79	0.00
WBC, 10^−3^ µL	5.7 ± 1.0	5.5 ± 1.0	−0.2	6.0 ± 1.9	6.5 ± 2.8	+0.5	(1,17) = 0.875	0.36	0.049
Haemoglobin, g/dL	14.15 ± 1.25	13.36 ± 1.87	−0.79	14.29 ± 0.99	12.95 ± 1.62	−1.34	(1,17) = 0.414	0.53	0.025
Haematocrit, %	0.41 ± 0.03	0.38 ± 0.05	+0.03	0.41 ± 0.03	0.39 ± 0.05	−0.02	(1,17) = 0.140	0.75	0.006
MCV, fL	90.47 ± 4.52	89.35 ± 4.78	−1.12	89.19 ± 3.14	88.31 ± 2.40	−0.88	(1,17) = 0.226	0.64	0.014
MCH, pg	30.81 ± 1.78	31.14 ± 2.00	+0.33	30.88 ± 1.75	29.27 ± 1.63 ^c^	−1.61	(1,17) = 7.190	0.016 *	0.310
MCHC, g/dL	33.66 ± 1.95	34.85 ± 1.52	+1.19	34.59 ± 0.87	33.14 ± 1.33 ^c^	−1.45	(1,17) = 5.426	0.033 *	0.250
RDW, μm	12.65 ± 0.70	12.52 ± 0.69	−0.13	12.89 ± 0.44	13.83 ± 2.34	+0.94	(1,17) = 1.897	0.19	0.106
Platelets, 10^−6^ µL	204.6 ± 36.2	202.9 ± 40.4	−1.7	194.5 ± 49.1	208.5 ± 56.6	+14.0	(1,17) = 0.957	0.34	0.053
Lymphocytes, % WBC	36.3 ± 7.2	34.5 ± 6.5	+2.2	34.8 ± 7.1	36.2 ± 8.1	+1.4	(1,17) = 1.023	0.33	0.057
Monocytes, % WBC	5.7 ± 1.4	7.0 ± 2.5	+1.3	5.7 ± 1.5	6.0 ± 1.2	+0.3	(1,17) = 1.344	0.26	0.073
Granulocytes, % WBC	57.9 ± 7.2	58.1 ± 6.1	+0.2	59.4 ± 7.5	57.7 ± 8.4	−1.7	(1,17) = 0.204	0.66	0.012
MPV, fL	8.1 ± 0.6	7.9 ± 0.8	−0.2	8.4 ± 1.2	7.9 ± 0.9	−0.5	(1,17) = 0.254	0.62	0.015
Serum Ferritin, ng/mL	117.6 ± 107.0	149.2 ± 103.6	+31.6	235.7 ± 90.3	249.9 ± 113.6	+14.2	(1,11) = 0.004	0.95	0.000

^a^ Original means and standard deviations, i.e., without adjustment for covariate. ^b^ ES = effect size. ^c^ Significant difference within group between pre- and post-intervention testing. *η*_p_^2^ = 0.01 (small effect), *η*_p_^2^ = 0.09 (medium effect), *η*_p_^2^ = 0.25 (large effect) [30]. * ANCOVA significant difference at *P* < 0.05. ^c^ Significant difference within group between pre and post-intervention. Abbreviations: RBC = red blood cells, WBC = white blood cells, MCV = mean corpuscular volume, MCH = mean corpuscular haemoglobin, MCHC = mean corpuscular haemoglobin concentration, RDW = red blood cell distribution width, % WBC = relative to total white blood cell count; MPV = mean platelet volume.
